# Case Report: A rare case of intramedullary spinal cord abscess with brain abscess caused by Klebsiella pneumoniae underwent surgical intervention

**DOI:** 10.3389/fsurg.2024.1338719

**Published:** 2024-02-27

**Authors:** Wenjuan Zhang, Xiangyu Guo, Xuejun Xu, Bing Deng

**Affiliations:** ^1^ Graduate School of Zunyi, Zunyi Medical University, Zunyi, Guizhou Province, China; ^2^ Department of Neurosurgery, Chengdu Second People’s Hospital, Chengdu, Sichuan Province, China

**Keywords:** intramedullary spinal cord abscess, brain abscess, Klebsiella pneumoniae, surgery, case report

## Abstract

**Background:**

Intramedullary Spinal Cord Abscess (ISCA) is an uncommon infectious disease of the central nervous system. Since its first report in 1830, there have been very few documented cases associated with it. Here, we present a case of ISCA with cerebral abscess caused by Klebsiella pneumoniae.

**Case presentation:**

A 55-year-old male patient presented with head and neck pain, fever, and left limb weakness for 5 days. The diagnosis of ISCA with brain abscess caused by Klebsiella pneumoniae was confirmed through sputum culture, cerebrospinal fluid gene test, pus culture, and magnetic resonance imaging (MRI) as well as computerized tomography (CT) scan. The patient had a history of pulmonary tuberculosis and old tuberculous foci were observed in the lung. Initially considering tuberculosis as the cause due to unclear etiology at that time, anti-tuberculosis treatment was administered. However, due to rapid deterioration in the patient's condition and severe neurological dysfunction within a short period of time after admission, surgical intervention including incision and drainage for intramedullary abscess along with removal of brain abscess was performed. Subsequent postoperative follow-up showed improvement in both symptoms and imaging findings.

**Conclusion:**

Early diagnosis of central nervous system (CNS) abscess coupled with prompt surgical intervention and administration of appropriate antibiotics are crucial factors in preventing disease progression and reducing mortality rates.

## Background

Intramedullary Spinal Cord Abscess (ISCA) is a rare infectious disease of the central nervous system, which has high mortality and neurological complications before the introduction of antibiotics and surgery due to its non-specific clinical manifestations and imaging features (1, 2). With the application of antibiotics, advanced neuroimaging examinations, and surgical treatment, the overall mortality rate has significantly improved from 90% in 1944 to 4% in 2009 (3). The etiological agents responsible for ISCA are diverse and have been extensively investigated in previous studies (2). Klebsiella pneumoniae is a gram-negative opportunistic pathogen that can cause various infectious diseases such as liver abscesses, endophthalmitis, meningitis, brain abscesses, pyogenic arthritis, etc. (4, 5). However, ISCA caused by Klebsiella pneumoniae is exceedingly rare and have not been previously documented. We report a case of ISCA with cerebral abscess caused by Klebsiella pneumoniae.

## Case presentation

The patient, a 55-year-old male, presented with head and neck pain, fever, and left limb weakness persisting for 5 days, accompanied by a peak temperature of 39.4°C. Subsequently, he developed progressive difficulties in rolling over and lower back pain. Additionally, the patient had a history of tuberculosis 10 years ago.

Upon admission, the patient presented with basic vital signs including a blood pressure of 105/75 mmHg, a pulse rate of 100 beats per minute, a respiration rate of 15 breaths per minute, and an average body temperature of 38.2°C. Physical examination revealed an alert and cooperative mental state, neck percussion tenderness, forced head positioning, partial left-sided neck involvement, grade 3 muscle strength in the left upper limb and grade 5- muscle strength in the left lower limb along with mild abnormal gait. The patient exhibited hypoalgesia below the knee on the left lower limb, tendon hyperreflexia, and positive pathological signs. Muscle strength and sensation in the right limb were within normal limits.

**Figure 1:**
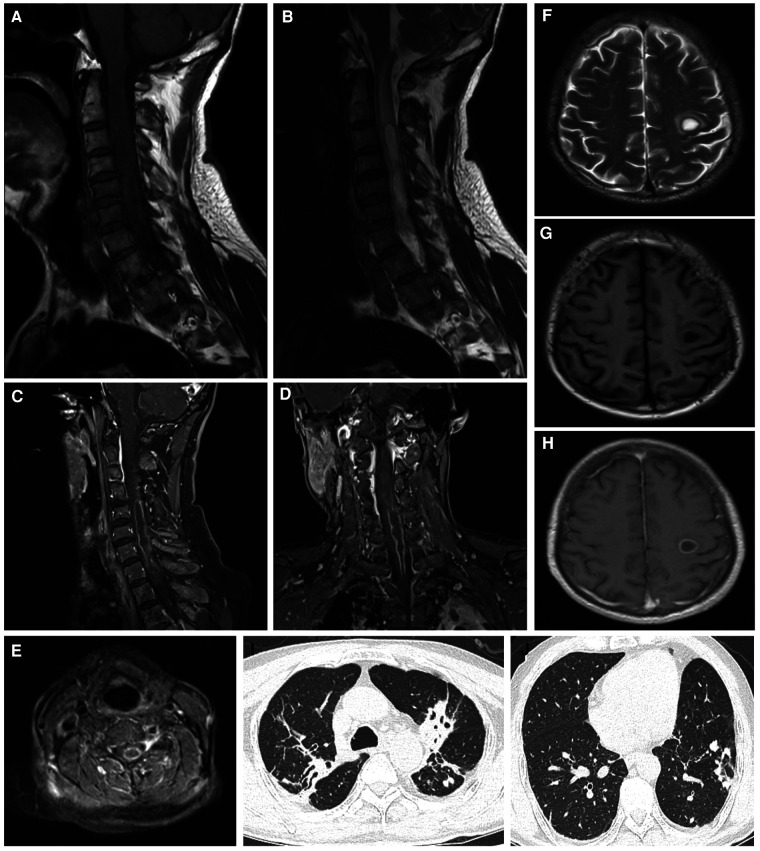
The spinal cord was imaged using MRI, including sagittal views in T1WI (**A**), T2WI (**B**), and contrast-enhanced T1WI (**C**) additionally, coronal views in contrast-enhanced T1WI were obtained (**D**), along with axial views in contrast-enhanced T1WI (**E**) furthermore, the brain was examined using MRI, specifically axial views in T1WI (**F**), T2WI (**G**), and contrast-enhanced T1WI images were acquired as well (**H**). Chest CT scans revealed suspicious tuberculosis foci (**I**, **J**). MRI, magnetic resonance imaging; CT, computerized tomography; T1WI, T1-weighted imaging; T2WI, T2-weighted imaging.

The initial laboratory findings of the patient were as follows: the white blood cell (WBC) count was 9.32 × 109/L, with a neutrophil percentage of 80.2%. Cerebrospinal fluid (CSF) analysis revealed a WBC count of 495.60 × 106/L. In terms of CSF biochemistry, the total protein level measured at 616 mg/L and glucose level at 2.3 mmol/L. Revised: The patient's initial laboratory results showed a white blood cell (WBC) count of 9.32 × 109/L, with a neutrophil percentage of 80.2%. Analysis of cerebrospinal fluid (CSF) indicated a WBC count of 495.60 × 106/L, while CSF biochemistry revealed levels of total protein measuring at 616 mg/L and glucose measuring at 2.3 mmol/L. Results from cerebrospinal fluid culture, tuberculosis bacillus smear, general bacterial smear, special stain, and tuberculosis antibody tests all yielded negative results; similarly, no positive results were obtained from blood culture either. MRI of the spinal cord showed long nodules with low-signal T1-weighted and high-signal T2-weighted MRI in the level between cervical vertebrae 2 to thoracic vertebrae 1 with edge intensification observed on contrast-enhanced T1-weighted MRI images. Similarly lesion in left frontal lobe with circular intensification and edema observed in the surrounding brain tissue on contrast-enhanced T1-weighted MRI images. Chest CT scan revealed the presence of emphysema, chronic infection in both lungs, and evidence of prior tuberculosis infection (Figure 1). Abdominal examination did not identify any significant intraperitoneal lesions.

After admission, the patient received initial treatment with ceftriaxone and meropenem for anti-infection purposes. In consideration of the potential presence of active tuberculosis, a comprehensive oral anti-tuberculosis regimen comprising isoniazid, rifampicin, and pyrazinamide was administered for several days. Subsequently, there was a sudden exacerbation of left limb hemiplegia (left upper limb muscle strength grade 0; left lower limb muscle strength grade 3; decreased sensation in the left knee joint). However, normal movement and sensation were observed in the right limb. Further sputum culture revealed Klebsiella pneumoniae as the causative agent while metagenomic next-generation sequencing (mNGS) analysis of cerebrospinal fluid confirmed Klebsiella pneumoniae as the pathogenic bacteria responsible for infection. Due to concerns regarding rapid disease progression potentially involving high cervical spinal cord and even medulla oblongata regions that could impact respiratory and circulatory function, an approved surgical intervention plan was implemented involving multi-point incision and drainage to eliminate cervical and thoracic intramedullary abscesses.

**Figure 2:**
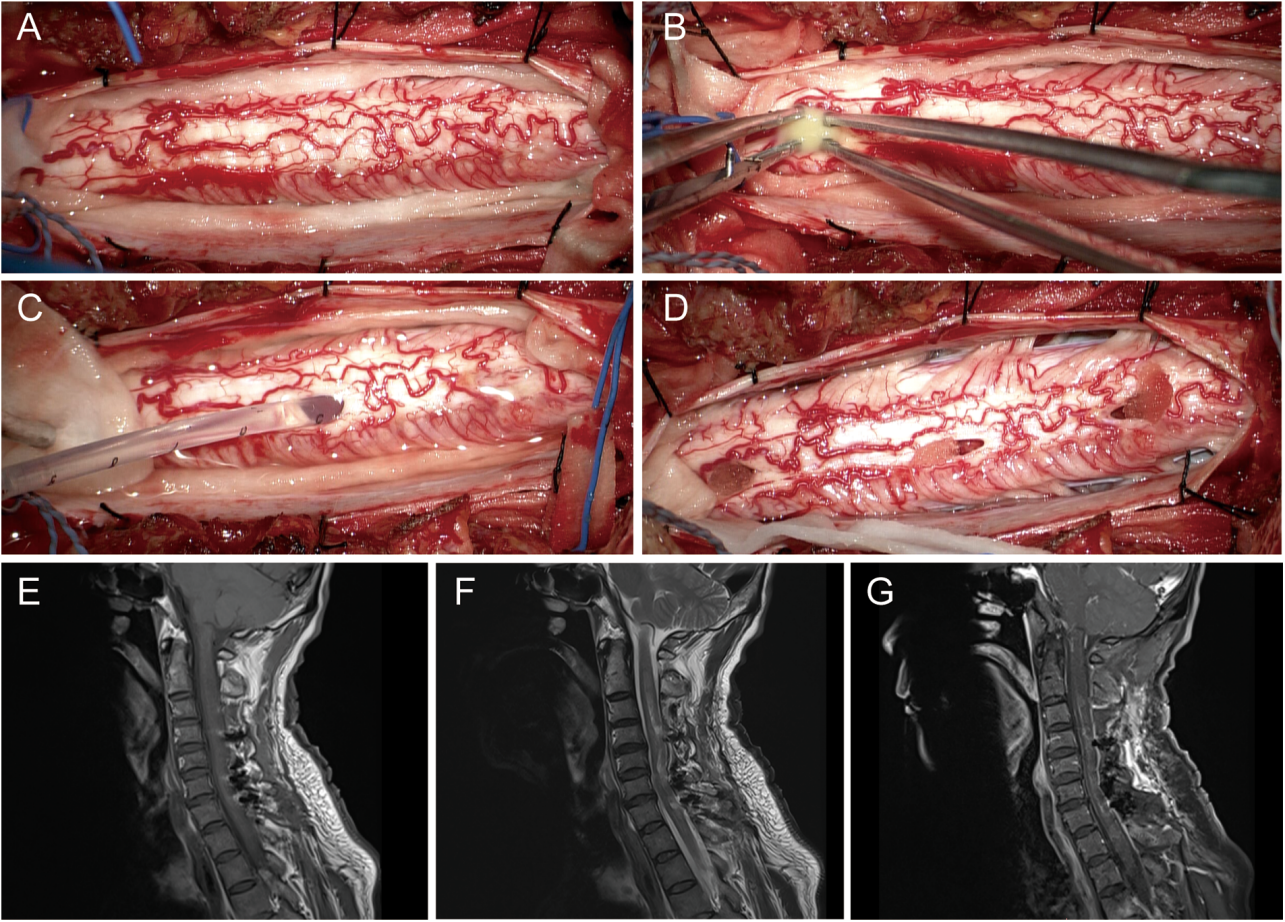
The surgical procedure for spinal cord surgery, which involves exposure (**A**), incision (**B**), drainage (**C**), and hemostasis (**D**); post-operative MRI images of the spinal cord are presented in T1WI (**E**), T2WI (**F**), and contrast-enhanced T1WI (**G**) MRI, magnetic resonance imaging; T1WI, T1-weighted imaging; T2WI, T2-weighted imaging.

During the surgical procedure, a purulent cavity measuring approximately 10 cm in length extended from the second cervical vertebra to the first thoracic vertebra. The pus cavity appeared yellow and white, with no discernible separation. Post-operative pus culture revealed an infection caused by Klebsiella pneumoniae, which exhibited sensitivity to most antibiotics tested (Figure 2). Anti-inflammatory treatment was administered using meropenem and linezolid, while anti-tuberculosis treatment was discontinued due to insufficient evidence. Following spinal cord surgery, muscle strength in the left upper limb improved to grade 3, muscle strength in the left lower limb improved to grade 4, and muscle strength in the right limb remained normal.

**Figure 3:**
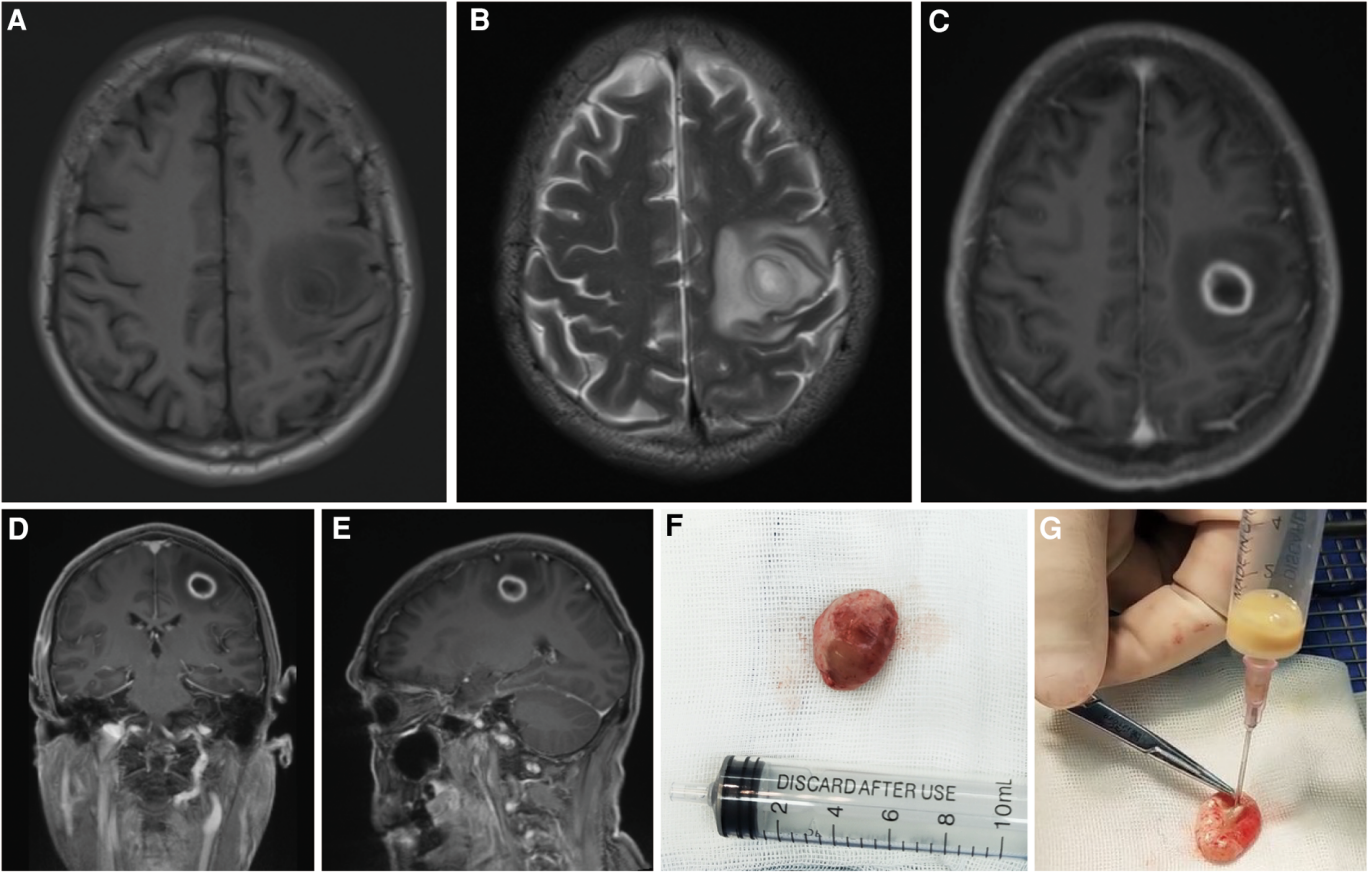
Brain MRI, including axial views in T1WI (**A**), T2WI (**B**), and contrast-enhanced T1WI (**C**), as well as coronal (**D**) and sagittal (**E**) views in contrast-enhanced T1WI, revealed the progressive nature of the left frontal lobe lesion. Unbroken cerebral abscess (**F**) and its pus (**G**) MRI, magnetic resonance imaging; T1WI, T1-weighted imaging; T2WI, T2-weighted imaging.

Three weeks after spinal cord surgery, the patient experienced an abrupt onset of muscle weakness in the right upper limb (muscle strength: right upper limb, grade 3; right lower limb, grade 5). The head MRI revealed an enlargement of the lesion located at the left frontal lobe, accompanied by aggravated surrounding edema. The surgical indication was evident, and a complete removal of the abscess without rupture of its wall was achieved during excision. Dissection of the abscess exhibited a thickened wall and yellowish pus within the cavity, resembling that found in the spinal cord, and pus culture yielded negative results (Figure 3). Following brain surgery, administration of antibiotic drugs (meropenem and linezolid) continued for ten days, with a total antibiotic course duration lasting approximately 5 weeks. After the second operation, the muscle strength of the right upper limb briefly decreased to grade 0. Prior to discharge, there was recovery observed in right upper limb muscle strength to grade 4.

## Discussion and conclusions

The intramedullary abscess of the spinal cord is an uncommon infectious disease affecting the central nervous system, which can lead to varying degrees of permanent nerve function impairment and even pose a threat to the patient's life. Byrne et al. found that 36% of intramedullary abscesses mainly involved the cervical cord, 36% involved the conus, and 29% involved the thoracic cord, and the specific symptoms were related to the site of infection (6). The infection mechanism of ISCA can be categorized into hematogenous dissemination, local spread (such as in proximity to the site of infection), and direct inoculation (resulting from spinal trauma, surgical procedures, congenital fistulae, etc.) (1). The time interval from the initial onset of symptoms to diagnosis can be categorized into three distinct phases: acute, subacute, and chronic (7). Acute symptoms typically resolve within a week, during which patients may manifest the classical triad of ISCA (fever, pain, and impaired neural function) (1).

Klebsiella pneumoniae, a Gram-negative opportunistic pathogen, colonizes the gastrointestinal tract and nasopharynx in humans. During immunosuppression, this bacterium can disseminate from the nasopharynx to other tissues or enter the bloodstream, leading to infection (4, 8). In this case, the sputum culture, pus culture, and mNGS all identified Klebsiella pneumoniae. The application of mNGS in clinical laboratories for unbiased culture-independent diagnosis is increasingly noteworthy (9, 10). CNS infections often manifest insidiously and can be accurately diagnosed using mNGS applied to CSF or biopsy specimens (11–13). We hypothesized that the patient might have acquired Klebsiella pneumoniae infection in the CNS as a result of partial destruction of paravertebral tissues caused by tuberculosis, followed by subsequent dissemination of the infection to adjacent tissues and CSF.

While the causative agent remained elusive, initial treatment involved a combination of anti-infective and anti-tuberculosis drugs. Despite this conservative approach, the disease progressed rapidly, causing severe neurological dysfunction. Prompt decompression surgery became crucial to alleviate symptoms. Multi-point abscess incision, irrigation, and drainage have proven effective in managing such abscesses. Three weeks after surgery, despite receiving appropriate antibiotics for the identified pathogen and showing no signs of infection spread, the patient experienced sudden muscle loss in their right upper limb. This unexpected development prompted surgical resection of the brain abscess. Postoperative antibiotics continued, and the patient's muscle strength transiently dropped to grade 0 in the right upper limb before gradually recovering over a week. Currently, their muscle strength has improved to grade 4.

When ISCA is clinically suspected, enhanced MRI examination of the corresponding site can be performed. Typical MRI findings of spinal cord and brain abscess include hypointensity on T1WI, hyperintensity on T2WI, and peripheral enhancement (1, 14). In cases where MRI fails to provide a definitive diagnosis, lumbar puncture can be employed as an adjunctive diagnostic tool for further elucidation (15). There is a dearth of guidelines pertaining to the diagnosis and treatment of ISCA, and although some patients exhibit improvement with antibiotic therapy alone, antibiotics primarily target smaller abscesses associated with ISCA (3, 16). For patients with rapidly deteriorating conditions suitable for surgery, early surgical intervention is recommended. Pus culture and drug sensitivity testing can provide further guidance for antibiotic therapy. The optimal duration of antibiotic treatment remains a subject of controversy. However, it is recommended to administer intravenous antibiotics for at least 4–6 weeks (1), and our patients received intravenous antibiotics for more than 4 weeks after surgery. In general, early implementation of drainage and prompt administration of intravenous antibiotics are indicative of a favorable prognosis (17). Surgical drainage within 5 days of symptom onset significantly improves neurological outcomes compared to conservative treatment or delayed drainage (17, 18).

Intracerebral abscess caused by Klebsiella pneumoniae is an extremely rare condition, and the clinical and imaging findings lack specificity. Early diagnosis of CNS abscess, along with prompt surgical intervention and administration of appropriate antibiotics, are crucial in preventing disease progression and reducing mortality rates. It should be noted that normal white blood cell count and blood culture results do not exclude the possibility of ISCA. Due to variations in pathogens, infection mechanisms, sites, and disease duration, typical clinical manifestations, imaging features, and laboratory results may not always be present; therefore, clinicians should remain vigilant regarding its occurrence.

## Data Availability

The original contributions presented in the study are included in the article/Supplementary Material, further inquiries can be directed to the corresponding author.
